# Obesity-Related Fatty Acid and Cholesterol Metabolism in Cancer-Associated Host Cells

**DOI:** 10.3389/fcell.2020.600350

**Published:** 2020-11-27

**Authors:** Ying Ye, Xiaoting Sun, Yongtian Lu

**Affiliations:** ^1^Department of Oral Implantology, Shanghai Engineering Research Center of Tooth Restoration and Regeneration, School and Hospital of Stomatology, Tongji University, Shanghai, China; ^2^Department of Medical Oncology, Shuguang Hospital, Shanghai University of Traditional Chinese Medicine, Shanghai, China; ^3^Department of Ear Nose Throat (ENT), Second People’s Hospital of Shenzhen, First Affiliated Hospital of Shenzhen University, Shenzhen, China

**Keywords:** cancer-associated host cells, metabolism, fatty acid oxidation, cholesterol metabolism, tumor microenvironment

## Abstract

Obesity-derived disturbances in fatty acid and cholesterol metabolism are linked to numerous diseases, including various types of malignancy. In tumor cells, metabolic alterations have been long recognized and intensively studied. However, metabolic changes in host cells in the tumor microenvironment and their contribution to tumor development have been largely overlooked. During the last decade, research advances show that fatty acid oxidation, cholesterol metabolism, and lipid accumulation play critical roles in cancer-associated host cells such as endothelial cells, lymph endothelial cells, cancer-associated fibroblasts, tumor-associated myeloid cells, and tumor-associated lymphocytes. In addition to anti-angiogenic therapies and immunotherapy that have been practiced in the clinic, metabolic regulation is considered another promising cancer therapy targeting non-tumor host cells. Understanding the obesity-associated metabolism changes in cancer-associated host cells may ultimately be translated into therapeutic options that benefit cancer patients. In this mini-review, we briefly summarize the lipid metabolism associated with obesity and its role in host cells in the tumor microenvironment. We also discuss the current understanding of the molecular pathways involved and future perspectives to benefit from this metabolic complexity.

## Introduction

The global obesity pandemic affects most high-income and middle-income countries, and it is associated with an increased incidence of certain types of cancer (Swinburn et al., 2011; Demark-Wahnefried et al., 2012). Obese adipocytes could release fatty acids (FAs), lipoproteins, hormones, and growth factors into extracellular space and circulation. It is recognized that adipocytes provide fuel and triggers for the metabolic reprogramming in changing tumor behaviors (Nieman et al., 2013).

**FIGURE 1:**
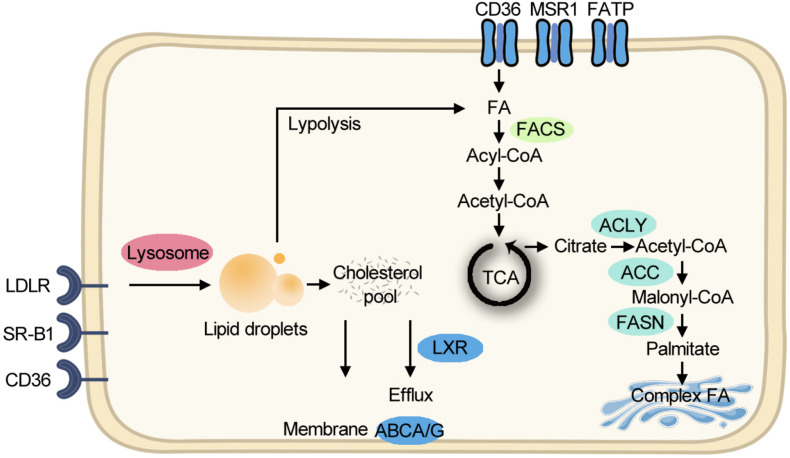
Simplified scheme of major lipid metabolic pathways. Exogenous FAs are taken up for FAO and energy production. Exogenous cholesterol-rich lipoproteins are imported for building membranes. *De novo* lipogenesis starts from exported citrate and makes complex FA in the Golgi body. FA, fatty acid; FAO, fatty acid oxidation; TCA, tricarboxylic acid cycle; CoA, coenzyme A; ACLY, ATP citrate lyase; ACC, acetyl-CoA carboxylase; FASN, fatty acid synthase; FACS, fatty acyl-CoA synthetase; ABCA/G, ATP-Binding Cassette A/G; LXR, liver X receptor; LDLR, low-density lipoprotein receptor; SR-B1, the scavenger receptor, class B type 1; MSR1, macrophage scavenger receptor 1; FATP, fatty acid transport proteins.

Although aerobic glycolysis is the dominant metabolic paradigm, cancer cells exploit lipid and cholesterol to meet their unlimited energy demands. In some types of cancer, lipid-dependent metabolism becomes a prominent pathway for energy production (Caro et al., 2012). Cancer cells obtain lipids by taking up the exogenous lipids and *de novo* synthesis of endogenous lipids. Free fatty acids (FFAs) are taken up through FA translocase CD36, FA transport proteins (FATPs)/SLC27A, and fatty acid-binding proteins (FABPs) for mitochondrial oxidation and energy production, while cholesterol-rich lipoproteins are taken up by receptors such as the low-density lipoprotein receptor (LDLR). Cholesterol can facilitate membrane microdomain formation, which can initiate tumor growth (Oneyama et al., 2009). Other than membrane composition, cholesterol is a precursor for bile acids and steroid hormones which can initiate cancer progression (Attard et al., 2009). For *de novo* lipogenesis, citrate is exported from the mitochondrion as a substrate, and ATP citrate lyase, acetyl-CoA carboxylase (ACC), and fatty acid synthase (FASN) sequentially promote FA production for further synthesis of triacylglycerols, cholesterol esters, and phospholipids. Compared with healthy tissues that prefer to use circulating lipids, tumor cells express a significant amount of FASN protein (Cai et al., 2015). Clinically, the first-in-class FASN inhibitor currently is under phase II trial and shows antitumor potential (Falchook et al., 2017). The lipid metabolism pathway is summarized in Figure 1.

Although the lipid metabolism and cholesterol metabolism in cancer cells *per se* have received substantial amount of interest over the past two decades, it needs to be considered that the tumor microenvironment (TME) is modulated by complex signaling networks from malignant cells and multiple other components, including vascular cells, stromal fibroblasts, inflammatory cells, and blood cells. Constantly exposed to various growth factors controlling tumor angiogenesis, lymphatic growth, stromal fibroblast expansion, and inflammation, TME becomes favorable for tumor growth, metastasis, and drug resistance. Whether obesity-derived FA and cholesterols can stimulate these host non-malignant cells is largely overlooked. There are still challenges to be faced in understanding how host cell lipid metabolism facilitates tumor development and in bringing the drugs that target cancer-associated host cell metabolism to the clinic.

## Obesity-Related Circulating FFA and Cholesterol Levels

In 1960, researchers found that most obese individuals have elevated FFA levels in serum (Gordon, 1960). FFAs from highly saturated fat consumption or dysregulated lipolysis can induce various responses, including insulin resistance, inflammation, lipotoxicity, and endothelial dysfunction (Pleiner et al., 2002; Jiao et al., 2011; Oh et al., 2018). Furthermore, FFAs exert pathophysiological functions through free fatty acid receptors (Vangaveti et al., 2010).

During obesity, dyslipidemia is a classic hallmark with reduced high-density lipoprotein cholesterol (HDL-C) levels and increased levels of small, dense low-density lipoprotein (LDL) particles, circulating triglycerides (TGs), or both (Kathiresan et al., 2006). Besides genetic dyslipidemia, lifestyle with excessive dietary intake of total calories, saturated fat, cholesterol, and trans fats are the leading cause for the increased rates of cholesterol synthesis and reduced expression of LDLR (Glatz and Katan, 1993).

## Lipid Metabolism in Endothelial Cells and Lymphatic Endothelial Cells

Tumor cell-derived angiogenic factors induce neovascularization in TME. Although tumors utilize similar mechanisms as normal growing tissues, tumor vasculatures are generally malformed with a high degree of disorganization, lack of clear separation between arterioles and venules, lack of pericyte coverage, and high permeability (Cao, 2009). Compared with the quiescent state of endothelial cells (EC) in healthy tissues, the angiogenic features of tumor vessels require a proliferative and migratory EC state, which is the basis of antiangiogenic drugs, such as bevacizumab. This sprouting state is recently shown driven by metabolic switches in EC.

It is reported that glycolytic flux is doubled under proliferative status in EC, and the glycolysis activator 6-phosphofructo-2-kinase/fructose-2,6-bisphosphatase 3 (PFKFB3) is the key enzyme for EC glycolysis (De Bock et al., 2013). Under physiological status, shear stress blocks PFKFB3-related metabolism in EC via flow-sensitive transcription factor (Doddaballapur et al., 2015). In contrast, PFKFB3 is upregulated in pathological angiogenesis and PFKFB3 blockade shows the antiangiogenic effect (Schoors et al., 2014). Although proliferating EC is considered to rely on glycolysis, it exerts a metabolic paradigm shift to fatty acid oxidation (FAO) upon glucose deprivation (Dagher et al., 2001). Indeed, CPT1α-guided FAO stimulates EC proliferation (Schoors et al., 2015), and inhibition of CPT1α in ECs impairs vessel stability (Patella et al., 2015). Interestingly, in proliferating EC, FAO is not providing additional ATP but is used for *de novo* synthesis of nucleotides (Schoors et al., 2015). It is reported that obesity-related hormone leptin promotes FAO in ECs by increasing CPT1α activity, suggesting obesity not only provides the fuel but also is capable of triggering host cell metabolism (Yamagishi et al., 2001). Moreover, under proliferation, EC increases FA uptake and the expression of FABP4 (Elmasri et al., 2009). FABP4 inhibition leads to a marked increase of FAO in tumoral EC and decreases tumor angiogenesis (Harjes et al., 2017). It is reported that vascular endothelial growth factor (VEGF)-B is involved in modulating FA uptake in EC (Hagberg et al., 2010). Whether VEGF-B blockade inhibits tumor angiogenesis requires further validation.

Emerging evidence suggests that cholesterol levels may regulate angiogenesis. Elevated circulating cholesterol level promotes tumor angiogenesis, and cholesterol uptake-blocking agent ezetimibe significantly inhibits tumor angiogenesis (Solomon et al., 2009). Cholesterol efflux from ECs to HDL reduces lipid rafts, interferes with VEGFR2 signaling, and inhibits angiogenesis (Fang et al., 2013). Activation of endothelial liver X receptors (LXRs), regulators of cholesterol homeostasis, reduces tumor angiogenesis by impairing the compartmentation of VEGFR2 (Noghero et al., 2012). Moreover, cholesterol trafficking is a potential target for blocking angiogenesis in TME (Lyu et al., 2017). Of note, VEGF-B is reported to impair LDLR recycling and reduce cholesterol uptake in EC. Its role in TME needs additional investigation (Moessinger et al., 2020).

The role of FA synthesis in tumor ECs remains incompletely understood. It is reported ACC regulates EC migration, and FASN is critical for vessel sprouting. ACC inhibition shifted the phospholipid composition of EC membranes and reduced membrane fluidity, filopodia formation, and migratory capacity (Glatzel et al., 2018). FASN knockout elevates malonyl-CoA levels, causing malonylation of mTOR and impairment of pathological angiogenesis (Bruning et al., 2018).

Intratumoral and peritumoral lymphatics are essential for lymph metastasis of tumor cells. However, the role of metabolism in lymphatic endothelial cells (LECs) is largely unknown. It is reported that FAO is crucial for lymphatic development through epigenetic regulation of lymphatic transcription factor PROX1 (Wong et al., 2017). The role of FA synthesis on the lymphatic network and tumor-induced lymphangiogenesis is still unknown. It seems like FASN is needed for the growth and maintenance of LECs, and FASN inhibitor reduces LEC migration and tumor lymph metastasis (Bastos et al., 2017).

It is reasonable to speculate that EC metabolism in TME is context-dependent (Li et al., 2019a). Single-cell RNA sequencing studies revealed extensive heterogeneity of metabolic gene expression signatures between ECs from different tissues (Li et al., 2019b). Tumor type may determine the metabolism pattern of ECs. Moreover, in TME, malignancy-derived various cytokines may disrupt EC metabolic switch. To target EC metabolism as an antiangiogenic therapy in TME, it is necessary to consider the impact of tumor type and tumor-derived cytokines. Hypoxia may be another parameter regulating EC metabolism. ECs, especially sprouting ECs, have to establish functional vessels for avascularized tissues. Under this status, it requires ECs to have a functional metabolism under hypoxia. In tumors, this need becomes even stronger. Whether obesity-derived FA and LDL can affect this unique metabolism requires further investigation. For LECs metabolism in TME, limited studies prevent us from understanding it in detail. We do know FAO and FASN are involved in the regulation of lymphangiogenesis. Further studies will be required to explore the mechanism and to understand the significance.

## Lipid Metabolism in Cancer-Associated Fibroblasts

Healthy fibroblasts can become activated during tumorigenesis. In general, cancer-associated fibroblasts (CAFs) promote tumor progression by secreting tumor-supporting factors, acting as a barrier to immune surveillance, and facilitate tumor cell migration (Yang et al., 2016b; Sahai et al., 2020). Of note, fibroblasts can exhibit phenotype heterogeneity; depletion of fibroblasts accelerates pancreatic cancer, suggesting CAFs may restrain tumor growth (Ozdemir et al., 2014). Multiple studies demonstrate fibroblast subsets in TME with differential abilities to affect tumor progression (Sahai et al., 2020). Understanding the metabolism of CAFs has potential importance for revealing the complexity of CAFs and their interactions with cell compartments in TME.

Several studies described metabolic features of CAFs, and majority of them focus on glucose and glutamine metabolism. Transforming growth factor (TGF)-β- or platelet-derived growth factor (PDGF)-induced CAFs switch from oxidative phosphorylation to aerobic glycolysis to meet the requirements of extracellular matrix (ECM) production (Zhang et al., 2015). It should be noted that studies on CAF lipid metabolism are quite limited. However, the lipid metabolism and fate determination of fibroblasts in other tissues can provide us with enlightenment. In TME, ECs may convert into a fibroblast-like state, known as endothelial-to-mesenchymal transition (EndoMT) (Platel et al., 2019). It is reported that FAO is a negative regulator for EndoMT (Xiong et al., 2018). In skin fibroblasts, peroxisome proliferator-activated receptors (PPARs) signaling that regulates FA uptake and oxidation promotes a catabolic phenotype by enhancing ECM internalization and lysosomal degradation (Zhao et al., 2019). The CD36-expressing fibroblasts transplantation improved skin elasticity and reduced ECM deposition in mice (Zhao et al., 2019). In general, FA uptake and FAO reduce fibrogenesis and proliferation in fibroblasts. This view warrants validation in CAFs.

Cholesterol levels may also regulate fibrogenesis and fibroblast activation. However, the existing evidence is insufficient and contradictory. In cardiac fibroblasts, LXR agonist prevents TGF-β-induced collagen synthesis and α-smooth muscle actin expression (Cannon et al., 2015). Another group reported that LXR inverse agonist suppresses fibrosis in non-alcoholic steatohepatitis *in vivo*, suggesting opposing effects of LXR signaling on regulating fibrogenesis (Griffett et al., 2015).

Interestingly, CAFs are capable of synthesizing and transferring lipids to neighboring cells (Santi et al., 2015). Higher FASN activity in CAF identified its role as FAs supplier in breast cancer (Lopes-Coelho et al., 2018). A recent study using lipidomic profiling showed that CAFs provide fatty acyl, long-chain, and unsaturated FAs for colorectal cancer tumor cell development (Gong et al., 2020). There is still a lack of evidence of lipid synthesis changing CAF phenotypes.

In other types of fibroblasts, FAO may reduce fibrosis and fibrogenesis. If this result can be replicated in TME, metabolic regulation in CAF may have antitumor potential. It is worth noting that upon CAF activation, ECM overexpression is not completely negative for tumor treatment. Type I collagen reduction, together with a significant decrease in tumor tissue stiffness, promotes tumor metastasis (Ozdemir et al., 2014). This work raises a warning for blunting CAF for fibrogenesis using FAO. The other point is that in skin fibroblast, FAO did not downregulate glycolysis *in vivo*, which means regulation is not made through a classic metabolic switching. It is necessary to verify this phenomenon in the TME. In general, due to the lack of understanding of CAF metabolism and the essential impact of CAF on the TME, research on CAF metabolism should become the next focus in this field. Once enough evidence is provided, it may present exciting new therapeutic opportunities for the management of cancer.

## Lipid Metabolism in Tumor-Associated Myeloid Cells and Lymphocytes

Understanding immune cell metabolism in cancer is of growing significance in the past decade with the success of immunotherapy. In TME, infiltrated immune cells include tumor-associated macrophages (TAMs), dendritic cells (DCs), myeloid-derived suppressor cells (MDSCs), and several T cell subpopulations. Understanding the differential metabolic requirements of diverse immune cells helps us regulate the complex immune response in TME. Among various kinds of tumor-associated host cell types, the role of lipid metabolism in regulating immune cells has been most intensively studied. Knowledge from classic immunology and evidence collected in TME indicates the importance of immune cell lipid metabolism in tumor growth and provides novel therapeutics for cancer. Evidence has been well-documented in several comprehensive reviews (Geeraerts et al., 2017; Le Bourgeois et al., 2018; Giovanelli et al., 2019; Leone and Powell, 2020). For the balance of this article, here we briefly discuss lipid metabolism in immune cells as below.

TAMs account for the largest fraction of the myeloid infiltrate in solid malignancies, and they display heterogeneous transcriptional programs and phenotype plasticity (Cassetta et al., 2019). At the beginning of this century, alternatively activated macrophages are found to be prone to increase FAO (Vats et al., 2006). Lipids uptake via CD36 and upregulation of FAO supports reactive oxygen species (ROS), JAK1-STAT6 activation, and hence the protumor function (Zhang et al., 2018; Su et al., 2020). DCs regulate the balance between immunity and tolerance through selective activation by the triggers in TME. Generally, tolerogenic DCs rely on FAO for their energetic demands, increasing the tumor burden in mice (Malinarich et al., 2015). Msr1-mediated FA uptake reduces DCs’ capacity to process antigens (Herber et al., 2010). MDSCs play vital roles in TME, and their inhibition is critical for successful cancer immunotherapy. MDSCs prefer FAO over glycolysis as a primary source of energy, while treatment with FAO inhibitors improved antitumor immunity (Hossain et al., 2015). In general, induction of FAO primes myeloid cells in TME for an immunosuppressive phenotype.

For cholesterol metabolism, TAMs readily take up lipoproteins from dying cells and developed mechanisms for eliminating cholesterol from the cell. Cholesterol efflux in TAMs supports IL-4 signaling and promotes tumor progression (Goossens et al., 2019). For other myeloid cells, cholesterol metabolism promotes a protumor phenotype. LXR signaling inhibits CCR7 expression in DCs and their migration to tumor-draining lymph nodes (Villablanca et al., 2010). In contrast, cholesterol loading in LXR knockout CD11c^+^ cells promotes the antigen presentation (Ito et al., 2016). Under a high-fat diet, cholesterol acts on neutrophils via its metabolite, 27-hydroxycholesterol, and priming an immune-suppressive environment for tumor metastasis (Baek et al., 2017).

Dysregulation of key enzymes in lipid metabolism may lead to various problems such as lipid accumulation. FASN upregulation in tumor-associated myeloid cells stimulates PPARβ/δ and supports tumor cell invasion (Park et al., 2015). In support of this view, various lipolytic enzymes involved in intracellular lipid metabolism, including monoacylglycerol lipase, AB-hydrolase containing 5, epidermal fatty acid-binding protein, and adipocyte/macrophage fatty acid-binding protein, strongly affect TAM function and tumor progression (Rao et al., 2015; Miao et al., 2016; Hao et al., 2018; Xiang et al., 2018). In DCs, chaperone-binding oxidatively truncated lipids prevent the translocation of pMHC to the surface of DC and partially explained the DC attenuation (Veglia et al., 2017). In MDSCs, lipid accumulation reprograms MDSC to be highly immunosuppressive cells (Al-Khami et al., 2017). In general, a number of studies indicate that lipid synthesis and accumulation play an important role in the function of myeloid cells.

T cells can be divided into many subtypes and kill tumors directly or indirectly by synthesizing various biological molecules. In T cells, glycolysis is observed upon activation by the T-cell receptors (TCR) and costimulatory signals in TME (Chang et al., 2013). In contrast, FAO mainly provides energy for Treg and memory T cells (O’Sullivan et al., 2014; Miska et al., 2019). FAO blockade suppresses Treg population, while the addition of FAs promotes their differentiation (Michalek et al., 2011). Interestingly, PD-1 inhibits glycolysis and promoting FAO in T cells, and PD-1 blockade recovers T cell capacity (Patsoukis et al., 2015), suggesting the link between lipid metabolism and immune checkpoint. Of note, linoleic acid, a type of FA accumulated in fatty liver disease, causes more oxidative damage and mediates selective loss of intrahepatic CD4^+^ T lymphocytes (Ma et al., 2016), suggesting obesity triggers specific T cell response.

The role of cholesterol metabolism in T cells is controversial. T cell activation triggers simultaneous suppression of the LXR pathway for cholesterol transport and induction of the sterol regulatory element-binding protein (SREBP) pathway for cholesterol synthesis (Bensinger et al., 2008). In TME, increasing the cholesterol level in CD8^+^ T cells may induce exhaustion by endoplasmic reticulum (ER) stress (Ma et al., 2019). However, another group reported that cholesterol causes enhanced T-cell receptor clustering in CD8^+^ T cells and antitumor effect (Yang et al., 2016a).

FA synthesis supplies membrane materials for activated effector T cells. For example, SREBPs are crucial for CD8^+^ T cell expansion (Kidani et al., 2013), while blocking ACC1 restrains the formation of TH17 cells and promotes the development of Treg cells (Berod et al., 2014). In general, lipid metabolism is also important for maintaining the balance between effector T cells and Treg cells; studies on the effect of lipid metabolism on T cell function need to focus on the different T cell types.

**FIGURE 2:**
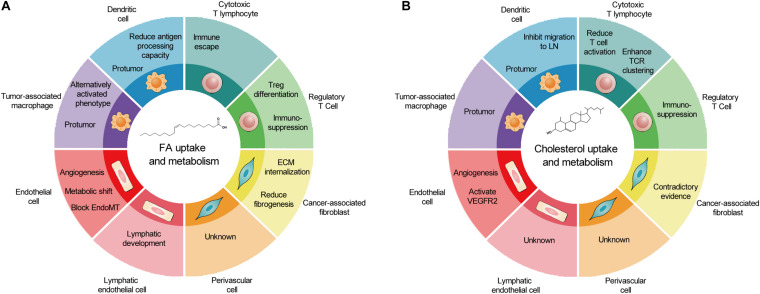
The outcomes of fatty acid and cholesterol metabolism in cancer-associated host cells. **(A)** In TME, FA metabolism promotes metabolic shift and angiogenesis in EC and LEC, reduces fibrogenesis, and generally induces protumor phenotype in immune cells. **(B)** In contrast, cholesterol metabolism promotes EC proliferation, induces protumor phenotype in immune cells, and produces contradictory effects in CAFs and cytotoxic T lymphocytes. Of note, several host cell types remain un-investigated. TME, tumor microenvironment; FA, fatty acid; EC, endothelial cell; LEC, lymphatic endothelial cell; CAFs, cancer-associated fibroblasts.

As a cell group with direct or indirect killing effect in TME, immune cells are attractive therapeutic targets for metabolic regulation. However, similar metabolism in activated antitumoral immune cells and tumor cells leads to competition between tumor development and antitumor immunity. In TME, glucose competition, hypoxia, and lactic acid secretion promote immunosuppressive phenotype in TAMs, DCs, and T cells. The accumulation of FAs caused by obesity may facilitate this process. The prerequisite of targeting lipid metabolism of immune cells to treat tumors is a better understanding of lipid metabolism in different cell types and its overall consequences. The effects of lipid metabolism on various types of cancer-associated host cells are summarized in Figures 2A,B.

## Conclusion

Targeting non-tumor host cells, such as antiangiogenic therapy and immunotherapy, has achieved significant effects in the clinical practice of cancer treatment. However, metabolic regulation for combating tumors has not yet become mainstream. Host cell metabolism in TME is significantly different from those in healthy tissues, and these differences provide opportunities to target host cell metabolism for treating tumors. Among the host cell populations in TME, immune cells are undoubtedly the most promising and the most studied cell group. Targeting the metabolism of suppressive immune cells or targeting effector cell metabolism to enhance tumor killing gives promising results in pre-clinical studies. However, similar evidence is lacking in other host cell types in TME. In this untouched research field, a series of studies are needed to understand obesity-related lipid metabolism and its influence on the host cells in TME. Moreover, future work should focus on the disruption of the TME on the metabolism of these host cells. The change of physical parameters in TME can increase angiogenesis, promote CAF activation, suppress the immune response, modulate drug resistance, and induce certain metabolic programs to support the malignancy. It is necessary to understand the molecular pathways involved in the host cell lipid metabolism to clarify what effect obesity has on this complex metabolic disruption and to benefit from this metabolic complexity.

## Author Contributions

YY generated the ideas and reviewed publications. YL and XS participated in discussions. YY and XS wrote the manuscript. All authors contributed to the article and approved the submitted version.

## Conflict of Interest

The authors declare that the research was conducted in the absence of any commercial or financial relationships that could be construed as a potential conflict of interest.
